# Are Older Adults More Risky Readers? Evidence From Meta-Analysis

**DOI:** 10.1037/pag0000522

**Published:** 2022-01-31

**Authors:** Jiaqi Zhang, Kayleigh L. Warrington, Lin Li, Ascensión Pagán, Kevin B. Paterson, Sarah J. White, Victoria A. McGowan

**Affiliations:** 1 Department of Neuroscience, Psychology and Behaviour, George Davies Centre for Medicine, University of Leicester; 2 Department of Psychology, Nottingham Trent University; 3 Academy of Psychology and Behavior, Faculty of Psychology, Tianjin Normal University

**Keywords:** aging, eye movements in reading, risky reading, Chinese

## Abstract

According to an influential account of aging effects on reading, older adults (65+ years) employ a more “risky” reading strategy compared to young adults (18–30 years), in which they attempt to compensate for slower processing by using lexical and contextual knowledge to guess upcoming (i.e., parafoveal) words more often. Consequently, while older adults may read more slowly, they might also skip words more often (by moving their gaze past words without fixating them), especially when these are of higher lexical frequency or more predictable from context. However, this characterization of aging effects on reading has been challenged recently following several failures to replicate key aspects of the risky reading hypothesis, as well as evidence that key effects predicted by the hypothesis are not observed in Chinese reading. To resolve this controversy, we conducted a meta-analysis of 102 eye movement experiments comparing the reading performance of young and older adults. We focused on the reading of sentences displayed normally (i.e., without unusual formatting or structures, or use of gaze-contingent display-change techniques), conducted using an alphabetic script or Chinese, and including experiments manipulating the frequency or predictability of a specific target word. Meta-analysis confirmed that slower reading by older compared to younger adults is accompanied by increased word-skipping, although only for alphabetic scripts. Meta-analysis additionally showed that word-skipping probabilities are unaffected by age differences in word frequency or predictability effects, casting doubt on a central component of the risky reading hypothesis. We consider implications for future research on aging effects on reading.

Eye movement research has helped build detailed accounts of visual, cognitive, and oculomotor control processes underlying skilled reading (Rayner, 1998, 2009), enabling the development of sophisticated computational models of eye movement control (Engbert et al., 2005; Reichle et al., 2003, 2006). This research has also provided insight into effects of normative aging on reading behavior (for reviews, see Gordon et al., 2015; Leinenger & Rayner, 2017; Moreno et al., 2019; Paterson et al., 2020). This is argued to show that older adults (65+ years) typically read more slowly than younger adults (18–35 years), by making more and longer fixations and more backward eye movements (regressions), consistent with slower and more disrupted text processing in older age. However, some studies of alphabetic languages additionally report that, compared to young adults, older adults are more likely to skip words (i.e., move their gaze past words without first fixating them) and make generally longer forward eye movements (saccades) in text (e.g., Rayner et al., 2006, 2009). Crucially, this pattern of eye movements differs from that observed for other groups of slower readers (e.g., less skilled young adult readers, readers with dyslexia; Ashby et al., 2005; Rayner, 1985), who skip words infrequently and make generally shorter forward saccades compared to skilled readers. Consequently, it is argued that this may represent a distinctive influence of aging on eye movement control in reading.

Slower and more disrupted reading by older adults generally is attributed to visual and cognitive declines in older age, including slower processing of visual and linguistic information (see, e.g., Gordon et al., 2015; Paterson et al., 2020). However, an influential account attributes differences in eye movement behavior to older adults adopting a qualitatively different reading strategy, as compared with young adults, to compensate for their slower processing of text (e.g., Rayner et al., 2006, 2009). According to this hypothesis, older adults attempt to maintain reading speeds at an acceptable level by employing a more “risky” reading strategy in which they make greater use of lexical and contextual knowledge, as well as partial information from the next word along (in parafoveal vision), to guess upcoming words. Crucially, the hypothesis predicts that, compared to young adults, older adults will skip words more often and so make generally longer forward saccades. However, as these guesses will sometimes prove wrong, older adults may also make regressions to reprocess text more often than young adults. As increased skipping rates are driven by the guessing of upcoming words, the hypothesis further predicts that older adults make greater use of information about a word’s lexical frequency (i.e., how often it is encountered in reading) and predictability (i.e., the degree to which a word is expected based on prior sentence context) to constrain these guesses. In support of this account, Rayner et al. (2006) demonstrated that incorporating these assumptions in a computational model of aging effects on reading (based on the E-Z Reader model of eye movement control; Reichle et al., 2003, 2006) could simulate the predicted pattern of slower reading times coupled with higher skipping rates and longer forward saccades. While not the only account available (see, e.g., Laubrock et al., 2006; Risse & Kliegl, 2011), the risky reading hypothesis has been central to many efforts to understand age-related changes in eye movement control.

In recent years, this hypothesis has faced two major challenges. First, the pattern of age differences predicted by the hypothesis has not been observed reliably across studies. In particular, several studies report no age differences in word-skipping or forward saccade length, despite observing slower reading by older adults (Choi et al., 2017; Whitford & Titone, 2016, 2017, 2019). Thus, while older adults in these experiments showed a typical slowdown in reading performance, this was not accompanied by a compensatory risky reading strategy. Moreover, a closer look at studies claimed to support the hypothesis reveals variability in effects, such that reported age differences in word-skipping and forward saccade length sometimes are not statistically reliable and even opposite to the predicted direction. For instance, the experiment Rayner et al. (2006) reported alongside their computational modeling of aging effects actually provides limited support for the hypothesis. This is because, although older participants skipped words numerically more often and made longer forward saccades than younger participants, the effects did not meet conventional standards for statistical reliability. Issues also arise when looking at studies assessing effects of word frequency and predictability. Such studies generally show larger effects for older adults in reading times on words (e.g., Kliegl et al., 2004; McGowan et al., 2014; Rayner et al., 2006, 2013; Steen-Baker et al., 2017; Whitford & Titone, 2017; Zhao et al., 2019, 2021). This is because, compared to young adults, older adults produce disproportionally longer reading times for words that are of lower frequency or less predictable, consistent with the view that lexical processing is slowed in older age. However, not all studies show these effects reliably, and even the original Rayner et al. (2006) does not find reliable interactions between age and predictability. In fact, a close inspection of the data suggests that for text presented normally, predictability effects are numerically larger for young than older adults in some measures. The situation is even more problematic when looking at effects on word-skipping. As noted above, the risky reading hypothesis proposes that older adults make greater use of lexical and contextual knowledge to guess (and so skip) upcoming words. This therefore predicts larger effects of word frequency and predictability on word-skipping for older compared to young adults. However, evidence for this is mixed, with several studies failing to observe this predicted pattern of effects (e.g., Choi et al., 2017; Whitford & Titone, 2017). The original Rayner et al.’s (2006) experiment also provides limited support for this claim, as older adults in this experiment produced larger frequency, but not larger predictability effects, in word-skipping probabilities compared to young adults.

The second challenge to the risky reading hypothesis arises from studies examining its cross-linguistic generalizability. Early studies examining age differences in reading, including Rayner et al.’s (2006) study, were focused on alphabetic scripts such as English and German. It is only more recently that studies have begun to examine whether such findings generalize to other languages, notably Chinese. Chinese is a logographic language consisting of box-like logograms, called characters, which can vary in visual complexity. Words are typically made up of one or two characters, although text in Chinese takes the form of an undifferentiated sequence of characters that does not use spaces or other visual cues to delineate word boundaries. Visual and linguistic demands in reading may therefore differ for Chinese as compared to alphabetic scripts like English or German, and so examining aging effects in Chinese reading may provide an effective test of the generalizability of the risky reading hypothesis. The findings from such studies show that Chinese is read much more slowly by older adults. However, contrary to the risky reading hypothesis, this slower reading is accompanied by reduced word-skipping and shorter forward eye movements (Wang, Li, Li, Xie, Chang, et al., 2018; Zang et al., 2016). In addition, while effects of word frequency and word predictability are longer in fixation times for words for older adults, studies to date provide no evidence for an age difference in the influence of these factors on word-skipping (Zang et al., 2016; Zhao et al., 2019, 2021). Accordingly, research with the Chinese script does not appear to support the risky reading hypothesis. However, whether this also means that effects reported for alphabetic scripts do not generalize to Chinese is unclear given the uncertainty over the reliability of these effects.

It should be clear from this overview that there exists significant variability in aging effects reported across different studies, even when ignoring the potential for cross-script differences. Several factors might contribute to this variability. First, aging research often uses small sample sizes (given difficulty recruiting older adults as participants) which may lack the statistical power required to detect effects reliably. Second, there is lack of uniformity in the age ranges investigated across studies, and in whether visual and cognitive assessments are administered to exclude participants with abilities outside the normal range. Consequently, differences may exist in participant profiles for studies showing and not showing specific patterns of aging effects. Third, evidence suggests that task demand characteristics can influence the likelihood of older adults engaging in different reading behaviors. Wotschack and Kliegl (2013) showed that age differences in word-skipping can be modulated by the frequency and difficulty of comprehension questions that follow sentence displays. In particular, whereas older adults in their study exhibited higher skipping than young adults when questions were easy and asked infrequently, this difference was reduced when questions were harder and asked frequently. Consequently, task demands may contribute to variation in effects reported across studies and may explain the absence of risky reading in key studies cited as counterevidence for this account (e.g., Choi et al., 2017; Whitford & Titone, 2017). Similarly, studies that use gaze-contingent methods to mask text or make surreptitious text changes (Choi et al., 2017; Whitford & Titone, 2016), manipulate text quality (McGowan et al., 2014; Rayner et al., 2013; Warrington et al., 2018) or use unusual sentence structures (Kemper et al., 2004), may impose additional visual, attentional, and working memory demands that encourage older adults to read more carefully than normal. As statistical analyses of key variables are often assessed in analyses that include these unusual text presentation conditions, age group effects that occur under normal reading conditions may be masked. Finally, while some studies report skipping rates across all words in a sentence, many report rates only for specific target words, which may provide a less sensitive measure as this will incorporate fewer data points. Word-skipping is also affected by other factors, including characteristics of the target word (e.g., word length) and words preceding it in a sentence (Kliegl et al., 2004; Paterson et al., 2013a; Rayner & McConkie, 1976). Consequently, analyses assessing word-skipping for only specific target words may miss overall differences in skipping rates across age groups. In light of these issues, determining aging effects from any individual study is unlikely to be fruitful.

Research in this area has nevertheless reached a level of maturity at which it may be possible to gain insights into aging effects using meta-analysis techniques. This was the approach taken in the present research, which used meta-analysis to examine aging effects across 102 separate eye movement studies conducted in either an alphabetic script or Chinese. Meta-analysis has the advantage of synthesizing findings from multiple studies to calculate an overall effect (e.g., Chan & Arvey, 2012; Verhaeghen, 2003), providing a quantitative assessment of the current state of play with respect to the literature. Eye movement studies are well suited to this task as they typically use similar designs, methods, and dependent variables and so have a high degree of comparability. Moreover, even studies that use gaze-contingent paradigms or manipulate text quality typically include a baseline condition in which text is presented normally. The present meta-analysis therefore examined eye movements for those conditions in experiments in which text was displayed as normal.

Our aim was to describe the current state of play regarding aging effects on eye movements in reading. We also aimed to provide a systematic assessment of the risky reading hypothesis, which seems timely considering recent studies that appear to contradict its predictions. Accordingly, we conducted a meta-analysis that focused on whether, compared to young adults, older adults (a) produce slower and more disrupted reading, that is, with longer reading times, more and longer fixations and more regressions across the whole of sentences, longer reading times on individual words and regions, and larger effects of word frequency and predictability in reading times for words; (b) make longer forward saccades and skip words more frequently, as characterized in the original Rayner et al.’s (2006) study; (c) exhibit a pattern consistent with increased guessing of upcoming words, by showing larger effects of word frequency and word predictability in skipping probabilities for words; and (d) whether findings for alphabetic languages generalize to Chinese.

## Method

### Transparency and Openness

The full data set and analytic code used are available (see Author Note). No materials were used. The number of studies and participants that contributed to each analysis are given in Tables 1–3. The gender and racial distribution of participants is unknown. All measures are reported. The study design, hypotheses, and analytic plan were not preregistered.[Table tbl1] [Table tbl2] [Table tbl3]

### Meta-Analysis

**Figure 1 fig1:**
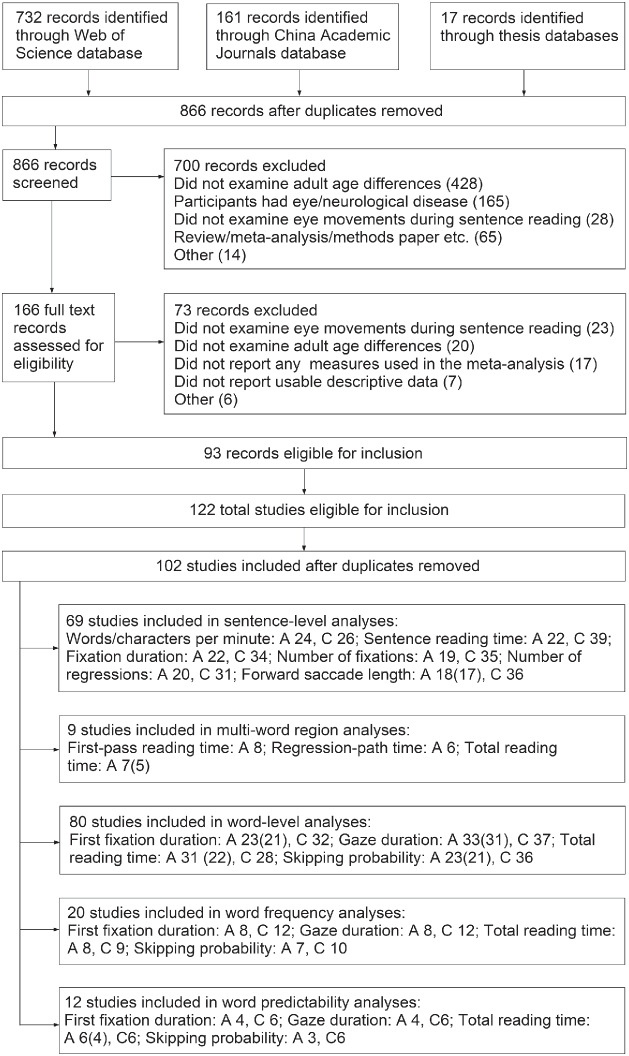
Flowchart of the Screening Process *Note*. Many excluded articles met multiple exclusion criteria, and so here we list only the first identified. The number of records identified through thesis databases refers only to those published in English, as theses published in Chinese are available through the China Academic Journals database. A = Alphabetic; C = Chinese. Where variance was not available for all studies, the number of studies for which this was available is given in brackets.

Relevant articles were identified using the search terms “eye movement*,” “reading,” and “age OR aging OR ageing OR older adult*” in Web of Science and the China Academic Journals database in August 2021. The same search terms were used to find unpublished studies in doctoral/master’s theses using major online thesis databases (ProQuest, EBSCO Open Dissertations, EThOS, OATD, DART, Deutsche Bibliothek, Theses Canada, Trove, and China Academic Journals database). Screening identified articles meeting the following inclusion criteria: (a) the article was published in English or Chinese; (b) the study examined eye movements during sentence reading in either an alphabetic language or Chinese; (c) both young (16–35 years) and older (60+ years) age groups were included, with descriptive statistics provided for each group; (d) participants were native readers of the studied language and data were available for first-language (L1) reading; (e) participants were reported to be free of eye or neurological disease; (f) at least one normal display condition was included, that is, with no unusual formatting, gaze-contingent display changes, highly unusual sentence structures (e.g., garden-path sentences), or concurrent task demands (e.g., working memory tasks); (g) descriptive statistics were available for sentences displayed normally for a least one measure examined in the meta-analysis. Individual experiments in multi-experiment articles/theses were considered separately unless using the same participants. Duplicates (e.g., where an experiment reported in a thesis was also published in article) were removed.^1^ Fifty-six published articles and 18 doctoral/master’s theses were included, totalling 102 individual experiments. Of these, 54 examined effects for alphabetic scripts (47 in English, 5 in German, 2 in French and English) and 48 for Chinese. None examined reading for both alphabetic languages and Chinese within the same study. Figure 1 shows a flowchart of the screening process. Note that in some cases, our inclusion criteria meant that not all measures reported in an experiment could be included (e.g., where means were reported separately for normal and nonnormal displays for some measures, but aggregated for others). A more comprehensive description of these studies (including participants’ cognitive, visual, educational, and reading abilities), as well as the full data set used for the meta-analyses, is included in the Online Supplement.[Fig fig1]

Adult age differences were examined across sentences (i.e., in measures incorporating eye movements across a single sentence), multiword regions (in measures incorporating eye movements across a region that contained more than one word, but less than a single sentence), and individual words in sentences (using measures incorporating eye movements for a single word). At the sentence level, we analyzed the following variables: words per minute reading rates for alphabetic scripts/characters per minute reading rates in Chinese, sentence reading time (ms), average fixation duration (ms), number of fixations, number of regressions, and forward saccade length (in letters/characters). For multiword regions, we analyzed the following variables: first-pass reading time (the sum of fixations on a region before the eyes move to the right or left, excluding cases where the region initially was skipped, in ms), regression-path duration (the sum of fixations from when the region is first fixated, to when the eyes move to its right, in ms), and total time (the sum of all fixations on the region, in ms). At the word level, we analyzed the following variables: first-fixation duration (the duration of the first first-pass fixation on a word, that is, excluding cases where the word was initially skipped, in ms), gaze duration (the sum of all first-pass fixations on a word, in ms), total reading time (the sum of all fixations on a word, in ms), and word-skipping probability (%). Additionally, adult age differences in word frequency and word predictability effects were examined using word-level measures. Only data from normal reading conditions were included (see the inclusion criteria above), averaged across different normal reading conditions (e.g., conditions containing a substituted short and long target word) where these were used.^2^ Word (or character) per minute reading rates were not always reported in the included studies. Where this reading rate was not reported, it was estimated by dividing sentence reading times by the average number of words/characters per sentence where this information was available. Note that variances cannot be reliably transformed using this method as sentence reading times typically have a skewed distribution (see Brysbaert, 2019). As very few variance measures were available, it did not prove possible to conduct meta-analysis for the reading rate data, and so we report only mean data for this measure.

Word-level analyses were restricted to single-word regions in sentences and averaged across words if multiple single-word regions were reported (e.g., where there was more than one target word in each sentence). For analyses of word frequency and word predictability, only studies which employed experimental manipulations were included, as corpus-based studies typically do not report descriptive data. For studies where more than two word frequency or predictability conditions were included (e.g., high, medium, low), we included the highest and lowest frequency conditions. WebPlotDigitizer (Rohatgi, 2018) was used to extract relevant values where descriptive statistics were available only in figures. Where a measure of variance was not reported, this was estimated by pooling standard deviations across the remaining data points (calculated separately for alphabetic scripts and Chinese) to avoid exclusion (Furukawa et al., 2006). For most studies, skipping rates were reported for individual target words in sentences, although skipping rates across all the words in a sentence were used where available to reduce variance (as this measure incorporates more data points).^3^ In some studies, skipping was reported as the number (rather than the percentage) of words skipped during first-pass reading; these numbers were converted into percentages when the average number of words per sentence was available. Where required, forward saccade length was converted from degrees of visual angle to number of letter/character spaces. The full data set is included in the Online Supplement.

Meta-analysis was conducted using the rma.uni function within the metafor package (Viechtbauer, 2010) in R (R Development Core Team, 2012, Version 4.0.1). A random-effects model was used as heterogeneity is likely due to variation in stimuli, methods, and language (for alphabetic scripts) across studies. Between-study variance was estimated using the restricted maximum likelihood estimator (Veroniki et al., 2016; Viechtbauer, 2005). Effect sizes were calculated using mean differences (Bond et al., 2003). For analyses of age differences in word frequency effects and word predictability effects, the size of the effect for each age group was entered into the meta-analysis. For reading time measures (where words that are low in word frequency or predictability are expected to have *longer* reading times than words that are high in word frequency or predictability), the size of the frequency effect was calculated as the mean of the low-frequency condition minus the mean of the high-frequency condition, and the predictability effect was calculated as the mean of the low predictability condition minus the mean of the high predictability condition. For word-skipping probability (where words that are low in frequency or predictability are expected to have *smaller* skipping rates than words that are high in frequency or predictability), the size of the frequency effect was calculated as the mean of the high-frequency condition minus the mean of the low-frequency condition, and the predictability effect was calculated as the mean of the high predictability condition minus the mean of the low predictability condition. This meant that all measures could be interpreted similarly, that is, with all frequency and predictability effects expected to be positive. The standard error of the mean difference was used as the measure of variance for these analyses. Forest plots were created using the forest function within the meta package (Balduzzi et al., 2019).

We first present sentence-level, multiword region, and word-level measures for alphabetic scripts, as adult age differences for these scripts form the basis of the risky reading hypothesis. To determine whether similar effects are observed in a nonalphabetic script, we then present sentence-level and word-level results for Chinese (note that none of the included Chinese studies assessed multiword regions). Finally, to establish whether effects obtained in alphabetic scripts might generalize to Chinese, we then present analyses examining all studies, with script type (alphabetic vs. Chinese) deviation coded (alphabetic coded as −0.5, and Chinese coded as 0.5) and included as a moderator variable. Analyses of script type were entirely between-study, as no studies examined the reading of both alphabetic languages and Chinese.

## Results

### Alphabetic Scripts

#### Sentence-Level Measures

**Figure 2 fig2:**
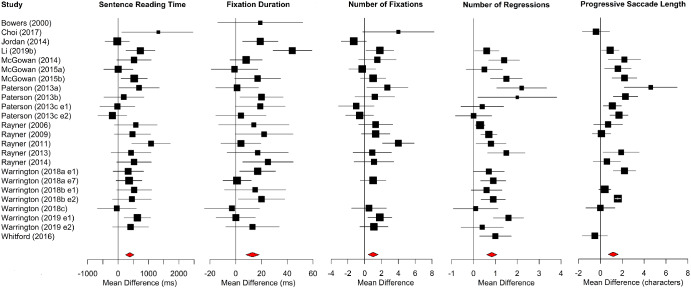
Forest Plots for Sentence-Level Measures in Alphabetic Languages *Note*. Age differences (i.e., the mean of the older adults minus the mean of the young adults) are plotted. Data points to the right of the line at 0 show larger values for older than younger adults, whereas data points to the left show the converse. For all studies, the horizontal lines intersecting each square represent the standard deviation, and the size of the square is proportionate to the weight of the study. Diamonds show overall effects. Study names across Figures 2–10 are given in full in the Supplemental Materials. See the online article for the color version of this figure.

**Figure 3 fig3:**
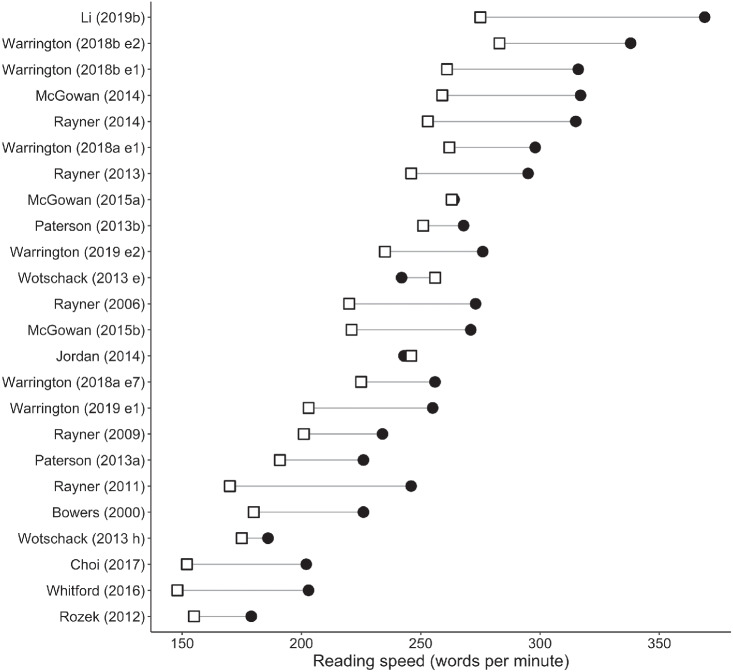
Mean Words Read Per Minute for Alphabetic Languages *Note*. Means for older adults are shown with open squares, and means for young adults are shown with closed circles.

Meta-analysis results for alphabetic languages are shown in Table 1. Forest plots for sentence-level measures are shown in Figure 2. The position of each data point represents the age difference for that study, that is, older adults’ mean minus young adults’ mean. Data points to the right of the line at 0 show larger values for older than younger adults, whereas data points to the left show the converse. Data for words per minute reading rates (which, as described above, did not have sufficient estimates of variance to conduct meta-analysis) are presented in Figure 3. Open squares and closed circles indicate means for older and young adults, respectively.[Fig fig2] [Fig fig3]

Older adults had longer sentence reading times, longer average fixations, more fixations, and more regressions, compared to young adults.^4^ Mean reading speeds were also slower for the older adults in almost every study. Importantly, however, older adults made longer forward saccades than young adults.

#### Multiword Region Measures

**Figure 4 fig4:**
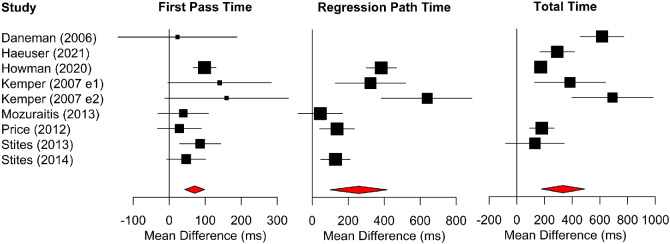
Forest Plots for Multiword Region Measures in Alphabetic Languages *Note*. Age differences (i.e., the mean of the older adults minus the mean of the young adults) are plotted. Data points to the right of the line at 0 show larger values for older than younger adults, whereas data points to the left show the converse. See the online article for the color version of this figure.

Forest plots for multiword region measures are shown in Figure 4. Older adults had longer first-pass reading times, regression-path times, and total reading times than young adults.^5^ [Fig fig4]

#### Word-Level Measures

**Figure 5 fig5:**
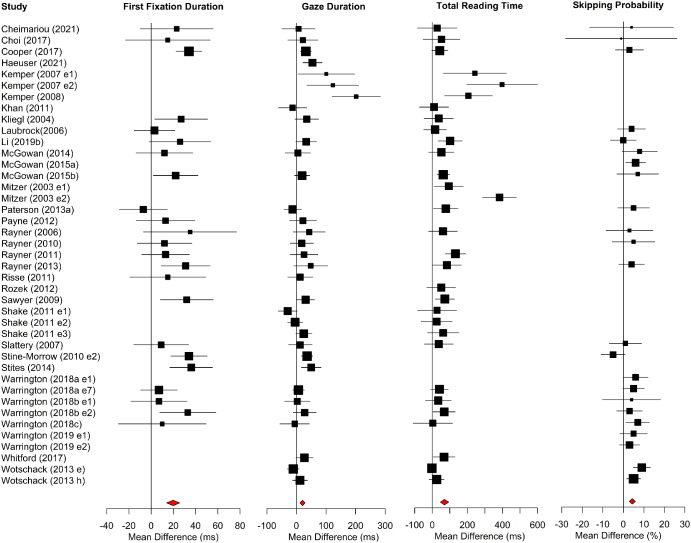
Forest Plots for Word-Level Region Measures in Alphabetic Languages *Note*. Age differences (i.e., the mean of the older adults minus the mean of the young adults) are plotted. Data points to the right of the line at 0 show larger values for older than younger adults, whereas data points to the left show the converse. See the online article for the color version of this figure.

Forest plots for word-level measures are shown in Figure 5. Older adults had longer first-fixations, gaze durations, and total reading times compared to young adults. Additionally, word-skipping rates were higher for the older adults.[Fig fig5]

Overall, the meta-analysis showed that older adults read more slowly than young adults by making more and longer fixations and more regressions, including spending longer reading individual words and multiword regions. However, forward saccades were longer and words were skipped more often by the older adults.

#### Word Frequency and Word Predictability

**Figure 6 fig6:**
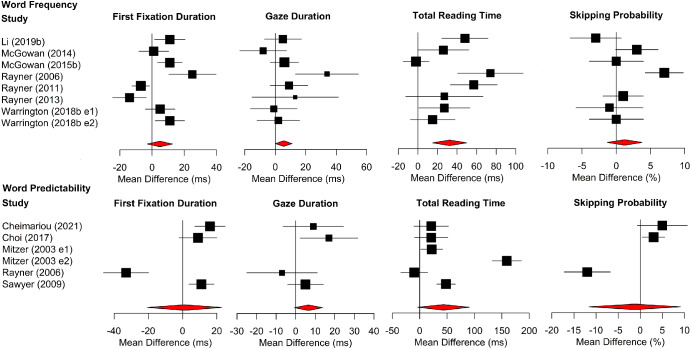
Forest Plots for Word Frequency Analyses (Top) and Word Predictability Analyses (Bottom) in Alphabetic Languages *Note*. The age difference (i.e., the mean of the older adults minus the mean of the young adults) in the size of the word frequency/predictability effect is plotted. For reading times measures, this is the mean for low-frequency/predictability words minus the mean for high-frequency/predictability words; for skipping probability, this is the mean of the high-frequency/predictability words minus the mean for low-frequency/predictability words. Data points to the right of the line at 0 therefore show larger frequency/predictability effects for older than younger adults, whereas data points to the left show the converse. See the online article for the color version of this figure.

Figure 6 shows forest plots for word frequency and word predictability effects. The position of each data point represents the age difference in the size of the word frequency or predictability effect (the frequency effect for older adults minus the frequency effect for young adults/the predictability effect for older adults minus the predictability effect for young adults). Accordingly, data points to the right of the line at 0 indicate that older adults had larger word frequency or predictability effects than young adults, whereas data points to the left indicate the converse.[Fig fig6]

Older adults had larger word frequency effects in gaze duration and total reading time. However, the age difference was not reliable for first-fixation durations or skipping probability. Age differences in the size of word predictability effects were not reliable for any measures.

Overall, there was evidence that, in comparison to young adults, older adults made disproportionately longer gaze durations and total reading times on words of low frequency compared to words of high frequency. However, there were no reliable age differences in the size of the predictability effects for reading times. Moreover, there was no indication of an age difference in the size of word frequency or word predictability effects in word-skipping. Accordingly, the meta-analysis presents no evidence that age differences in word-skipping are driven by older adults making greater use of lexical or contextual knowledge to guess (and so skip) upcoming words more frequently. We note, however, that this interpretation is based on only a small number of experiments (ranging from *n* = 3 for predictability effects on word-skipping to *n* = 8 for word frequency effects for the reading time measures).

### Chinese Script

#### Sentence-Level Measures

**Figure 7 fig7:**
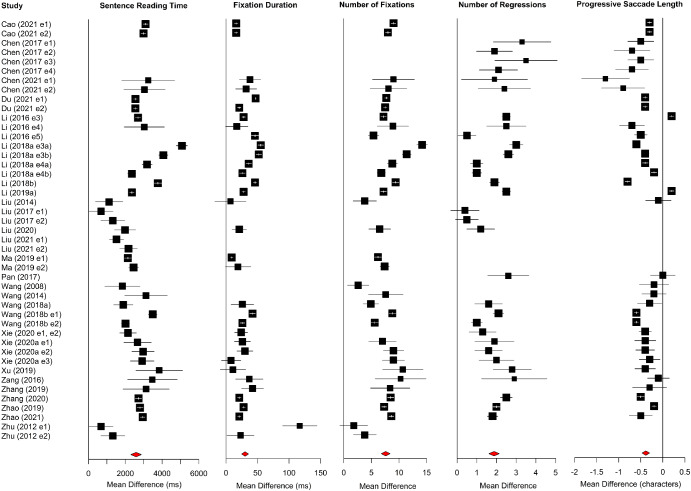
Forest Plots for Sentence-Level Measures in Chinese *Note*. Age differences (i.e., the mean of the older adults minus the mean of the young adults) are plotted. Data points to the right of the line at 0 show larger values for older than younger adults, whereas data points to the left show the converse. See the online article for the color version of this figure.

**Figure 8 fig8:**
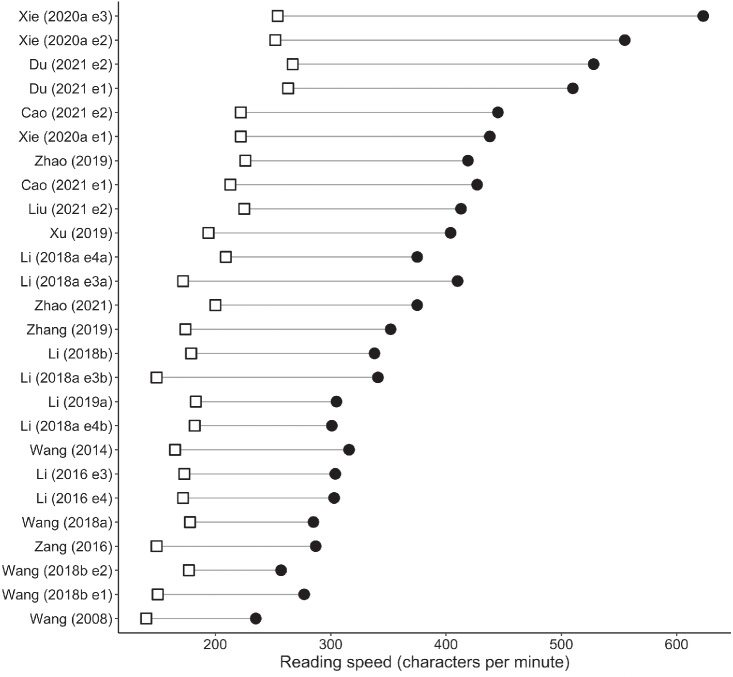
Mean Words Read Per Minute for Chinese *Note*. Means for older adults are shown with open squares, and means for young adults are shown with closed circles.

Meta-analysis results for Chinese are shown in Table 2. Forest plots for sentence-level measures are shown in Figure 7, and mean character per minute reading rates are shown in Figure 8. Older adults had longer sentence reading times, longer average fixations, more fixations and more regressions, compared to young adults. Unlike for alphabetic scripts, forward saccades were shorter for older than younger adults.[Fig fig7] [Fig fig8]

#### Word-Level Measures

**Figure 9 fig9:**
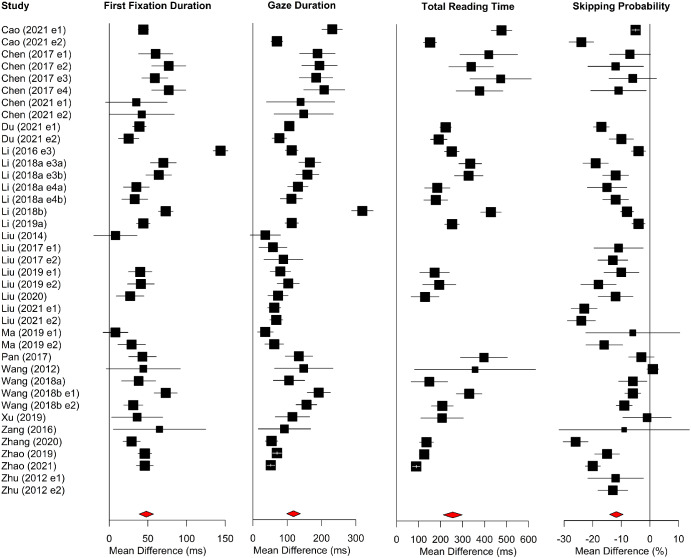
Forest Plots for Word-Level Region Measures in Chinese *Note*. Age differences (i.e., the mean of the older adults minus the mean of the young adults) are plotted. Data points to the right of the line at 0 show larger values for older than younger adults, whereas data points to the left show the converse. See the online article for the color version of this figure.

Forest plots for word-level measures are shown in Figure 9. Older adults had longer first-fixations, gaze durations, and total reading times compared to young adults. Unlike for alphabetic scripts, word-skipping rates were reduced for older relative to younger adults.[Fig fig9]

Overall, the results show that, similarly to alphabetic scripts, older adults read Chinese more slowly than young adults, by making more and longer fixations and more regressions, and spending longer reading individual words. However, forward saccades were shorter and skipping rates were reduced for older relative to younger adults.

#### Word Frequency and Word Predictability

**Figure 10 fig10:**
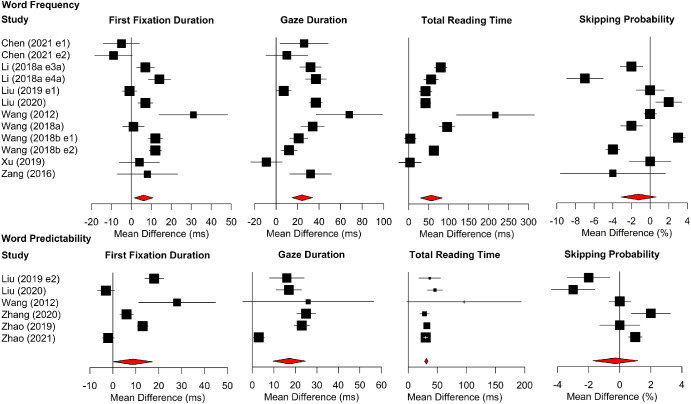
Forest Plots for Word Frequency Analyses (Top) and Word Predictability Analyses (Bottom) in Chinse *Note*. The age difference (i.e., the mean of the older adults minus the mean of the young adults) in the size of the word frequency/predictability effect is plotted. For reading times measures, this is the mean for low-frequency/predictability words minus the mean for high-frequency/predictability words; for skipping probability, this is the mean of the high-frequency/predictability words minus the mean for low-frequency/predictability words. Data points to the right of the line at 0 therefore show larger frequency/predictability effects for older than younger adults, whereas data points to the left show the converse. See the online article for the color version of this figure.

Figure 10 shows forest plots for the word frequency and predictability effects. Word frequency effects were larger for older adults in first-fixation durations, gaze durations, and total reading times. There was no age difference in the size of the word frequency effect in word-skipping probability.[Fig fig10]

Word predictability effects were larger for older adults in first-fixation durations, gaze durations, and total reading times. There was no age difference in the size of the word predictability effect in word-skipping probability.

As with findings for alphabetic scripts, word frequency effects were larger for older than younger adults in reading time measures. Unlike the data for alphabetic scripts, word predictability effects were larger for older than younger adults across all reading time measures. Critically, there was no age difference in the effects of word frequency or predictability on word-skipping, and so no evidence that older adults made greater use of lexical or contextual knowledge to skip upcoming words. As for the analyses of word frequency and predictability effects in alphabetic languages, we note that this interpretation is based on only a small number of experiments (ranging from *n* = 6 for all measures of word predictability effects to *n* = 12 for word frequency effects in first-fixation duration and gaze duration).

### Moderation of Age Effects by Script Type

Meta-analysis results for analyses that included both alphabetic languages and Chinese are shown in Table 3. Analyses that included both alphabetic and Chinese data showed that script type moderated age group effects for all sentence- and word-level measures. For sentence reading times, average fixation durations, number of fixations and regressions, and reading times on words, age group effects were in same direction for both script types, but larger for Chinese. Importantly, the direction of age effects differed for forward saccade lengths and word-skipping rates—for alphabetic scripts, forward saccades were longer and skipping rates were higher for older adults, whereas for Chinese, older adults’ saccades were shorter and skipping rates were lower relative to young adults.

Word frequency analyses that included both alphabetic and Chinese data produced main effects of age group in first-fixation durations, gaze duration, and total reading times. The moderating effect of script type was reliable for gaze duration, as age differences in the size of the word frequency effect for this measure were larger for Chinese. There was no significant moderating effect of script type for first-fixation durations or total reading times. The size of the word frequency effect in skipping probabilities did not differ as a function of age group, with no moderating effect of script. Thus, even when alphabetic and Chinese data were pooled, there was no evidence of an age difference in the influence of word frequency on word-skipping.

Word predictability analyses that included both alphabetic and Chinese data showed that the word predictability effect was larger for older adults in gaze durations and total reading times, but not first-fixation durations or word-skipping probability. Finally, script type did not reliably moderate age differences in the predictability effect for any measures.

### Publication Bias

To assess the likelihood of publication and other biases, funnel plots were created using the funnel function in the meta package (Schwarzer, 2007). These are generated using variances from individual studies and so we excluded studies with pooled variance. As there were relatively few studies examining word frequency and predictability effects, funnel plots for these measures include data for both alphabetic languages and Chinese. The resulting plots can be found in the Online Supplement (Figures S1–S7). For each experiment (shown as filled circles), the age difference (older adults’ mean minus young adults’ mean) is plotted against the standard error. In the absence of bias, a symmetrical funnel-shaped pattern of filled circles should be observed, such that experiments with smaller standard errors (i.e., high precision) cluster toward the top of the graph and experiments with larger standard errors (i.e., low precision) scatter along the bottom. A visual inspection indicates lack of symmetry in several plots. There are many reasons why funnel plots are not symmetrical, including chance (Sterne et al., 2011). Nevertheless, we assessed the likely impact of this asymmetry using the trim and fill method within the metafor package (Viechtbauer, 2010) to trim studies that contributed to the asymmetry and fill in estimated missing studies (Duval & Tweedie, 2000). We then reran the meta-analyses. Note that the trim and fill function cannot be used with meta-analyses that include a moderator, and so we conducted these analyses separately for alphabetic languages and Chinese for sentence-level and word-level analyses, but across both script types for the word frequency and word predictability analyses. Age group effects were no longer reliable for number of fixations in alphabetic scripts (95% CI [−0.1, 1.3], *z* = 1.77, *p* = .077), and there were no longer significant age differences in the size of the word frequency effect for first-fixation duration (95% CI [−1, 8], *z* = 1.72, *p* = .085). However, all other measures produced the same direction and similar statistical significance as the original analyses. Notably, almost all unpublished studies showed the same direction of effects as published studies. Publication bias therefore did not appear to affect our findings or interpretations to any meaningful extent.

## Discussion

The present meta-analysis pooled data from 102 experiments to determine age differences in eye movement behavior. We found a clear pattern of aging effects across multiple studies, even though such effects were not observed in some individual studies. In addition, data from both alphabetic scripts and Chinese confirm that older adults read more slowly, by making more fixations and regressions, and spending longer fixating words compared to young adults. Older readers of alphabetic languages make longer forward saccades and skip words more often than young adults; however, older Chinese adults make shorter forward saccades and skip words less often. There is some evidence that older adults have larger word frequency and word predictability effects in reading times than young adults, however, neither script type showed evidence of an age group difference in the effects of these variables on word-skipping.

### Adult Age Differences in the Reading of Alphabetic Languages: Evidence of Risky Reading?

For alphabetic scripts, including English, older adults read more slowly, made more and longer fixations and more regressions, and spent longer reading individual words. Our meta-analysis also confirms that this slowdown in reading in older age is coupled with increased word-skipping and generally longer forward saccades. This aspect of our findings is consistent with the risky reading hypothesis as described by Rayner et al. (2006). Other aspects of our findings, however, were not consistent with the risky reading hypothesis. We described in the Introduction how the hypothesis predicts that older adults will make greater use of lexical and contextual knowledge to guess (and so skip) upcoming words. Contrary to this prediction, the meta-analysis showed no significant age group difference in effects of word frequency or predictability on word-skipping probabilities. Consequently, the evidence to date does not support this prediction and suggests that, contrary to the risky reading hypothesis, older adults do not make greater use of lexical and contextual knowledge to skip words more frequently. This conclusion is in line with several influential corpus-based studies that did not meet our inclusion criteria (Kliegl et al., 2004; Whitford & Titone, 2017). However, it is important to note that relatively few studies contributed to our analyses for word frequency and predictability, and so caution is needed in interpreting these findings. For analyses of alphabetic scripts, only seven studies contributed to the meta-analysis of effects of word frequency on skipping and the equivalent analyses for word predictability included just three studies. Our understanding of these effects would therefore benefit from more studies that investigate age difference in lexical prediction.

This leaves the question of why older adults exhibit higher word-skipping probabilities and longer forward saccades when reading alphabetic scripts. The meta-analysis suggests these effects are not moderated by a word’s frequency or predictability and so cannot be explained in terms of older adults making greater use of lexical or contextual knowledge to guess upcoming words (and so skip them more often). However, the effects might be compatible with an account in which older adults attempt to compensate for slower processing by guessing words identities based on only visual and orthographic information about upcoming words (e.g., word length, beginning letter information). Such an account may resonate with a simulation of aging effects by McGowan and Reichle (2018; based on the E-Z Reader model) which incorporated mechanisms that allowed for increased word-guessing (and therefore skipping) that was not linguistically driven. Another possibility is that older adults attempt to compensate for slower processing by using a skim-reading strategy in which they can skip words frequently while still obtaining the gist of the sentence meaning (e.g., Just & Carpenter, 1987; Masson, 1982; White et al., 2015). Such effects may also be compatible with the proposal that older adults are more likely to read “mindlessly,” and so often temporarily process text to only a superficial level (Wotschack & Kliegl, 2013). Finally, it is possible that the increased word-skipping is a consequence of poorer saccadic control by older adults, with their saccades more frequently overshooting words. Evidence from nonreading tasks points to deficits in saccadic control in older age (e.g., Irving et al., 2006; Nieuwenhuis et al., 2000; Peltsch et al., 2011; Warabi et al., 1984). However, while few reading studies have assessed saccadic control by older adults, those that have show that older adults can target their saccades toward words as effectively as young adults can during reading (Paterson et al., 2013a; Rayner et al., 2006). This and the other possible explanation outlined above clearly merit further investigation, and an examination of nonlinguistic influences on word-skipping has the capacity to provide fresh insights into the nature of aging effects on eye movement control in reading.

### Cross-Script Differences

The meta-analysis provided clear evidence for cross-script differences in aging effects, indicating that findings for alphabetic languages may not be generalizable to other scripts. We found that older adults read Chinese more slowly than young adults, by making more and longer fixations and more regressions. However, whereas aging effects for alphabetic scripts were characterized by increased word-skipping and longer forward saccades, for Chinese they involved skipping words infrequently and making shorter forward saccades compared to young adults.

Various explanations of these cross-script differences are possible. These include participant sampling differences across experiments with different scripts and also a specific age-related cost for Chinese. However, an explanation based on sampling differences seems unlikely given the care taken in matching young and older adult participants in most experiments, including in terms of participants’ years of education, reading experience, and vocabulary knowledge. There is therefore no indication from these studies that the young and older adult participants differ in terms of literacy (although it is worth noting that for both Chinese and alphabetic readers, matching for years of education may not fully capture variance in educational experience). Moreover, the finding from the present meta-analysis that age differences in effects of word frequency are broadly similar across alphabetic scripts and Chinese suggests that the young and older adults who participated in these experiments have similar language processing capabilities. One strong possibility is that the visual and cognitive demands associated with the Chinese script are a specific source of difficulty for older adult readers. Indeed, our analyses which included script type as a moderator indicated that age differences in sentence reading times were larger in Chinese than alphabetic languages, which is consistent with the notion that the reading of Chinese may be particularly challenging for older readers. These demands are likely to include visual processing difficulties associated with identifying complex characters (e.g., Li, et al., 2019; Zang et al., 2016), as well as cognitive processing costs involved in establishing boundaries between words (Li et al., 2015). Further work is required to more fully understand how the specific visual and cognitive demands of Chinese impact on different age groups of readers. Another possibility is that task demands or demand characteristics differ across studies of alphabetic scripts and Chinese. Both explanations are in line with other evidence showing that older adults read more slowly and more carefully when text is visually challenging or difficult to comprehend (McGowan et al., 2014; Rayner et al., 2013; Warrington et al., 2018; Wotschack & Kleigl, 2013). Furthermore, reading goals can modify eye movement behavior (Just & Carpenter, 1987; White et al., 2015), and such goals may differ across participant groups.

We note, however, that we did not find any studies that directly examined cross-linguistic age differences, and so presently our understanding of such effects is entirely based on comparisons of different studies. Such comparisons are limited by methodological differences across studies (e.g., stimuli characteristics). While direct cross-linguistic comparisons of eye movements in reading are inherently difficult (Liversedge et al., 2016), future studies which adopt this approach will be essential for understanding how the specific characteristics of different scripts may mediate aging effects.

### Age Differences in Word Frequency and Word Predictability Effects

The meta-analysis provided clear evidence for age group differences in the effects of word frequency on the processing of fixated words. This was because, compared to young adults, older adults made disproportionately longer reading times for low-frequency words relative to high-frequency words. This larger word frequency effect in older compared to young adults occurred for both the reading of alphabetic languages and Chinese. Coupled with the finding that older readers make generally longer fixations on words, these effects might be consistent with slower lexical processing in older age. This slower processing might be a consequence of cognitive decline. Alternatively, it may be because growth in the mental lexicon from a lifetime of exposure to written language can slow access to specific lexical items (e.g., Blanco et al., 2016; Ramscar et al., 2014) or because of increased competition from orthographic neighbors in older readers (Payne et al., 2020). Moreover, the direction of the effect is also unclear. It could represent a processing cost for lower frequency words or a processing benefit for higher frequency words. However, there was no indication that older adults made greater use of word frequency information than young adults to skip upcoming words.

The meta-analysis also provided some evidence of age group differences in the effects of word predictability. Older readers of Chinese had disproportionately longer reading times for low predictable compared to high predictable words in comparison to their younger counterparts. This indicates that young and older Chinese readers differ in their use of context when processing words. Older readers of alphabetic languages also showed numerically larger predictability effects than young adults in gaze durations and total reading times, although these findings did not reach conventional standards of statistical significance. Intriguingly, studies using event-related potentials (ERPs) have typically found that an ERP component (the N400) known to be sensitive to the contextual fit of a stimulus is smaller or delayed for older relative to younger adult participants, indicating that older adults might be less able to use context predictively (e.g., Federmeier & Kutas, 2005; Hamberger et al., 1995; Payne & Federmeier, 2018; Wlotko et al., 2012; Wlotko & Federmeier, 2012; and for a recent review, see Payne & Silcox, 2019). This conclusion is at odds with evidence from the present meta-analysis which indicates greater use of context by older than young adults, at least for the reading of Chinese. We note, however, that relatively few studies contributed to our analyses of word predictability effects. Nevertheless, an important goal for future research will be to reconcile the apparently divergent patterns of findings obtained when using ERP compared to eye movements methods.

### Recommendations for Future Studies

In this final section, we turn to a discussion of how our experience in performing the present meta-analysis might inform best practice in conducting and reporting eye movement studies of aging effects and inform directions future research. First, while the meta-analysis produced generally clear results, some discrepancies among studies were observed, particularly for alphabetic scripts. Some of this variability will be attributable to variations in stimulus material characteristics, for example, sentences that vary in length and/or complexity, multiword regions that differ in the number of words, or critical words that differ in length, frequency, or predictability. As noted in the Introduction, some between-study variance might be because of small sample sizes in some studies, differences in the profile of participant groups across studies, or use of experimental methods that promote more careful reading (e.g., use of frequent or difficult comprehension questions). We note that studies that have recently been cited as counterevidence for the risky reading strategy (e.g., Choi et al., 2017; Whitford & Titone, 2016) had slower reading speeds for both young and older adults than many other studies (see Figure 3), suggesting that participant or task characteristics may affect the direction of age effects. Such effects are worthy of further investigation.

Benefits may also be obtained from fuller assessment and reporting of the visual and cognitive capabilities of participant groups. Many studies (including from our laboratory) have not reported these variables comprehensively and so it is unclear whether some discrepancies between studies might be attributable to the inclusion of participants with visual or cognitive capabilities outside the normal range. Including better information about participant profiles will improve the comparability of study findings and advance understanding of how individual differences in visual and cognitive capabilities might affect reading performance. Improvements to current assessment methods should include more rigorous assessment of visual abilities, which decline markedly in older age and are affected by eye disease common to older adults (e.g., cataract, age-related macular degeneration; see Owsley, 2011). Studies of aging effects on reading often do not test visual abilities (for discussion, see McGowan et al., 2013) or else rely on self-report measures despite their unreliability (Laitinen et al., 2005). We therefore recommend that researchers conduct appropriate visual screening tests, ideally administered under the same viewing conditions and at the same viewing distance used in the experiment. Many studies also do not screen for unimpaired cognitive function (to exclude effects of neurological disease, such as dementia) or systematically assess factors that show change with age and that are likely to affect reading performance. We therefore also recommend the use of screening tools for cognitive dysfunction, as well as cognitive assessments (e.g., working memory, vocabulary) to assess for cross-group differences.

It also became apparent in conducting the meta-analysis that many studies (again, including some from our own laboratory) do not fully report all eye movement measures that are useful for assessing aging effects. In some cases, this was sufficiently serious that experiments, including several considered to be landmark studies in the field, violated our inclusion criteria. These included studies which either did not report raw means or variance or did not provide descriptive statistics separately for normal and nonnormal text displays (e.g., Kemper et al., 2004, 2006). Furthermore, there were wide discrepancies in measures reported within studies. For example, only 54 out of 102 studies reported data for forward saccade length. Future meta-analysis would therefore benefit from studies reporting a broader range of eye movement measures. These include sentence-level measures of reading time, number and duration of fixations, regression rate, and forward saccade length, even in experiments focused on word-level effects, to help establish the comparability of participant groups. We also recommend reporting skipping rates for all words in sentences, in addition to specific target words, to provide a more sensitive measure of aging effects on word-skipping probabilities. While this comprehensive reporting of measures may not have been feasible in the past, and journal editors may have encouraged the reporting of only those measures appropriate for hypothesis testing, it is now possible to provide this more comprehensive reporting of measures as Supplemental Materials or to make data available via online open access repositories. The wider availability of trial-by-trial and word-by-word data will be particularly valuable for efforts to examine another key prediction of the risky reading hypothesis that increased regression rates of older adults are a consequence of increased skipping. In general, we recommend the wider adoption of open science methods to assist future efforts to synthesize findings across studies.

Finally, we consider it important to note that investigations of aging effects on eye movements in reading conducted to date tend to be limited to the reading of isolated sentences by young (18–35 years) or older adults (65–80 years). Few studies have investigated reading multi-sentence texts (but see Payne & Stine-Morrow, 2012; Stine-Morrow et al., 2010; Whitford & Titone, 2017, 2019) although it will be important to assess whether effects in studies reviewed here, which principally concern the reading of sentences displayed across a single line, generalize to more typical reading situations. Similarly, few studies have investigated reading in middle-aged participants (but see Payne et al., 2020; Steen-Baker et al., 2017; Warrington et al., 2018), and to our knowledge, no studies have examined aging effects separately for young-old adults (60–69 years) and older-old adults (80+ years), who may have distinct cognitive profiles (Smith & Bates, 1993). Such studies will nevertheless be crucial for obtaining a detailed understanding of the changes that take place in cognitive and oculomotor mechanisms across the adult lifespan.

## Supplementary Material

10.1037/pag0000522.supp

## Figures and Tables

**Table 1 tbl1:** Meta-Analysis Results for Alphabetic Languages

		*n* participants		Effect of age			
Measure	*n* studies	Young	Older	Estimated mean	95% CI [lower, upper]	*z*	*p*
Sentence level							
Sentence reading time	22	417	404	385	[246, 524]	5.43	<.001
Fixation duration	22	403	390	13	[8, 18]	4.93	<.001
Number of fixations	19	367	354	1.0	[0.4, 1.6]	3.26	.001
Number of regressions	20	392	379	0.8	[0.6, 1.0]	7.41	<.001
Forward saccade length	18	332	319	1.2	[0.7, 1.6]	5.00	<.001
Multiword region							
First-pass reading time	8	208	213	71	[43, 98]	5.00	<.001
Regression-path time	6	160	160	258	[99, 416]	3.19	.001
Total reading time	7	177	178	334	[177, 490]	4.19	<.001
Word level							
First-fixation duration	23	597	544	20	[14, 26]	6.54	<.001
Gaze duration	33	856	811	21	[12, 29]	4.66	<.001
Total reading time	31	839	807	70	[46, 94]	5.66	<.001
Skipping probability	23	551	504	4	[3, 6]	5.71	<.001
Word frequency							
First-fixation duration	8	147	134	5	[−3, 13]	1.20	.231
Gaze duration	8	147	134	6	[0, 11]	1.97	.049
Total reading time	8	147	134	32	[15, 50]	3.62	<.001
Skipping probability	7	115	102	1	[−1, 4]	0.95	.343
Word predictability							
First-fixation duration	4	87	88	1	[−21, 23]	0.10	.917
Gaze duration	4	87	88	7	[−1, 14]	1.77	.076
Total reading time	6	143	144	43	[−4, 91]	1.80	.072
Skipping probability	3	66	67	−1	[−12, 9]	−0.24	.811

**Table 22 tbl2:** Meta-Analysis Results for Chinese

		*n* participants		Effect of age			
Measure	*n* studies	Young	Older	Estimated mean	95% CI [lower, upper]	*z*	*p*
Sentence level							
Sentence reading time	39	1,244	1,142	2,587	[2,299, 2,875]	17.61	<.001
Fixation duration	34	1,100	1,013	30	[25, 36]	10.99	<.001
Number of fixations	35	1,086	999	7.6	[6.8, 8.4]	18.70	<.001
Number of regressions	31	982	862	1.9	[1.6, 2.2]	13.08	<.001
Forward saccade length	36	1,093	999	−0.39	[−0.5, −0.3]	−8.82	<.001
Word level							
First-fixation duration	32	1,054	909	48	[39, 57]	10.04	<.001
Gaze duration	37	1,215	1,047	118	[98, 139]	11.42	<.001
Total reading time	27	886	738	255	[212, 299]	11.59	<.001
Skipping probability	36	1,148	984	−12	[−14, −9]	−9.79	<.001
Word frequency							
First-fixation duration	12	430	372	6	[1, 11]	2.55	.011
Gaze duration	12	430	372	24	[15, 34]	4.93	<.001
Total reading time	9	318	260	57	[29, 84]	4.07	<.001
Skipping probability	10	350	292	−1	[−3, 1]	−1.29	.197
Word predictability							
First-fixation duration	6	246	188	9	[0, 17]	1.99	.046
Gaze duration	6	246	188	17	[10, 25]	4.44	<.001
Total reading time	6	246	188	31	[28, 34]	21.77	<.001
Skipping probability	6	246	188	0	[−2, 1]	−0.38	.701

**Table 3 tbl3:** Meta-Analysis Results That Included Both Alphabetic Languages and Chinese

		*n* participants		Effect of age				Moderating effect of script			
Measure	*n* studies	Young	Older	Estimated mean	95% CI [lower, upper]	*z*	*p*	Estimated mean	95% CI [lower, upper]	*z*	*p*
Sentence level											
Sentence reading time	61	1,661	1,546	1,502	[1,302, 1,702]	14.71	<.001	2,171	[1,771, 2,571]	10.63	<.001
Fixation duration	56	1,503	1,403	22	[18, 26]	10.4	<.001	17	[9, 25]	4.07	<.001
Number of fixations	54	1,453	1,353	4.3	[3.7, 4.9]	14.21	<.001	6.5	[5.3, 7.7]	10.70	<.001
Number of regressions	51	1,374	1,241	1.4	[1.2, 1.6]	13.22	<.001	1.0	[0.6, 1.4]	4.90	<.001
Forward saccade length	54	1,425	1,318	0.4	[0.2,0.5]	5.31	<.001	−1.5	[−1.8, −1.3]	−10.79	<.001
Word level											
First-fixation duration	55	1,651	1,453	34	[27, 40]	10.44	<.001	29	[16, 41]	4.46	<.001
Gaze duration	70	2071	1858	71	[59, 83]	11.47	<.001	93	[68, 117]	7.46	<.001
Total reading time	58	1,725	1,545	164	[139, 189]	12.83	<.001	179	[129, 229]	7.00	<.001
Skipping probability	59	1,699	1,488	−4	[−6, −2]	−4.36	<.001	−16	[−19, −12]	−9.12	<.001
Word frequency											
First-fixation duration	20	577	506	5	[1, 10]	2.45	.014	1	[−7, 10]	0.34	.736
Gaze duration	20	577	506	15	[9, 22]	4.46	<.001	17	[4, 31]	2.55	.011
Total reading time	17	465	394	44	[28, 59]	5.53	<.001	21	[−10, 52]	1.36	.175
Skipping probability	17	465	394	0	[−2, 2]	−0.01	.990	−2	[−6, 1]	−1.53	.126
Word predictability											
First-fixation duration	10	333	276	5	[−5, 15]	1.05	.292	8	[−12, 28]	0.76	.448
Gaze duration	10	333	276	12	[5, 18]	3.64	.003	11	[−2, 23]	1.64	.100
Total reading time	12	389	332	41	[17, 66]	3.28	.001	−4	[−54, 45]	−0.17	.866
Skipping probability	9	312	255	−1	[−4, 2]	−0.43	.667	1	[−5, 7]	0.23	.821

## Footnotes

1 It is unclear whether the same data set is reported in Kliegl et al. (2004) and Laubrock et al. (2006), as although the text reports this to be the case the participant numbers are discrepant. Here we included both studies separately, although our results were not changed by removing either study.

2 For two studies, we averaged across conditions containing unequal amounts of data for skipping probability. As a precaution, we ran additional meta-analysis using data from the condition with the smallest age difference and observed the same pattern.

3 The same pattern of results was observed when running the meta-analysis using data for target words only.

4 Given inherent variability in sentence length across studies, we also conducted analyses of number of fixations per word and number of regressions per word. This was calculated by dividing means and standard deviations for each study by its average number of words (alphabetic studies) or characters (Chinese studies). For alphabetic studies, these analyses were carried out using data from 16 and 17 studies for number of fixations and regressions respectively, and for Chinese, these analyses were carried out using data from 21 and 16 studies respectively. Similar to the main analyses, older readers of alphabetic languages made more fixations per word (95% CI [0.05, 0.17], *z* = 3.91, *p* < .001) and more regressions per word (95% CI [0.06, 0.10], *z* = 7.37, *p* < .001) than young readers. Similarly, older readers of Chinese also made more fixations per character (95% CI [0.36, 0.44], *z* = 18.65, *p* < .001) and more regressions per character (95% CI [0.08, 0.11], *z* = 9.95, *p* < .001) than their younger counterparts.

5 As there was also considerable variability in the number of words per multiword region, we conducted analyses of first-pass reading times, regression-path times, and total reading times per word for these regions. This was calculated by dividing means and standard deviations for each study by its number of words per region. These analyses were carried out using data from 6, 4, and 6 studies respectively for first-pass reading times, regression-path times, and total reading times. Similar to the main analyses older readers made longer first-pass reading times per word (95% CI [15, 44], *z* = 4.07, *p* < .001), regression-path times per word (95% CI [47, 136], *z* = 4.05, *p* < .001), and total reading times per word (95% CI [65, 213], *z* = 3.66, *p* < .001).
