# Supplementary material for: Are Older Adults More Risky Readers? Evidence From Meta-Analysis
Source: Psychol Aging. 2022 Jan 31;37(2):239–59. doi: 10.1037/pag0000522 (PMC8867715; doi:10.1037/pag0000522)
Supplement: Supplementary file 1 [file PAG-2020-0942_Suppl.zip › Figure Legends for Supplementary Figures.docx]

Figure Legends

Figure S1. Funnel plots for the sentence-level measures in alphabetic languages.

Figure S2. Funnel plots for the sentence-level measures in Chinese.

Figure S3. Funnel plots for the multi-word region measures in alphabetic languages.

Figure S4. Funnel plots for the word-level measures in alphabetic languages.

Figure S5. Funnel plots for the word-level measures in Chinese.

Figure S6. Funnel plots for the word frequency analyses.

Figure S7. Funnel plots for the word predictability analyses.
